# Waste not, want not: Microsatellites remain an economical and informative technology for conservation genetics

**DOI:** 10.1002/ece3.8250

**Published:** 2021-10-25

**Authors:** Samantha S. Hauser, Giridhar Athrey, Paul L. Leberg

**Affiliations:** ^1^ Department of Biology University of Louisiana at Lafayette Lafayette Louisiana USA; ^2^ Faculty of Ecology and Evolutionary Biology Texas A&M University College Station Texas USA

**Keywords:** genomics, molecular markers, next‐generation sequencing, parentage, population structure, resistance surfaces

## Abstract

Comparisons of microsatellites and single‐nucleotide polymorphisms (SNPs) have found that SNPs outperform microsatellites in population genetic analyses, questioning the continued utility of microsatellites in population and landscape genetics. Yet, highly polymorphic markers may be of value in species that have reduced genetic variation. This study repeated previous analyses that used microsatellites with SNPs developed from ddRAD sequencing in the black‐capped vireo source‐sink system. SNPs provided greater resolution of genetic diversity, population differentiation, and migrant detection but could not reconstruct parentage relationships due to insufficient heterozygosities. The biological inferences made by both sets of markers were similar: asymmetrical gene flow from source sites to the remaining sink sites. With the landscape genetic analyses, we found different results between the two molecular markers, but associations of the top environmental features (riparian, open habitat, agriculture, and human development) with dispersal estimates were shared between marker types. Despite the higher precision of SNPs, we find that microsatellites effectively uncover population processes and patterns and are superior for parentage analyses in this species with reduced genetic diversity. This study illustrates the continued applicability and relevance of microsatellites in population genetic research.

## Graphical abstract

Comparisons of microsatellites and single‐nucleotide polymorphisms (SNPs) have found that SNPs outperform microsatellites in population genetic analyses, calling into the question the continued utility of microsatellites in population and landscape genetics. This study repeated analyses previously done using microsatellites with SNPs developed from ddRAD sequencing in the black‐capped vireo source‐sink system. Despite the higher precision of SNPs, we find that microsatellites effectively uncover population processes and patterns and are superior for parentage analyses in this species with reduced genetic diversity.

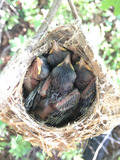

## 1 INTRODUCTION

Molecular markers allow us to answer an array of population genetic questions about gene flow (Edelaar & Bolnick, 2012; Hudson et al., 1992), parentage (García et al., 2002), and population structuring (Clark‐Cockerham & Weir, 1993; Narum et al., 2008). The toolbox of molecular markers has rapidly advanced in the last 30 years, progressing from allozyme markers to microsatellites to single‐nucleotide polymorphism (SNP) markers, each with progressively higher statistical power (Andrews & Luikart, 2014; Luikart et al., 2003; Morin et al., 2004). The higher resolution of microsatellite and SNP data has extended their use to landscape genetics (Sork et al., 2010), tests of adaptation and selection (Ahrens et al., 2018), hybridization (Toews et al., 2016), outbreeding and inbreeding depression (Steiner et al., 2013), and epigenetics (Harrisson et al., 2014). Of particular interest here is the relatively recent interdisciplinary field of landscape genetics that combines the theory and methods from landscape ecology and population genetics to study how landscape (or seascape) features affect population processes such as gene flow (Zeller et al., 2012). Landscape genetics bridges the gap between environmental factors and species’ responses, providing singular insights into ecological and evolutionary processes. Information gleaned from molecular markers offers crucial insights into application in conservation and management efforts.

Microsatellites and SNPs are the most commonly used markers for population genetic studies, each with pros and cons (Morin et al., 2009). Microsatellites are highly polymorphic, providing relatively high statistical power per locus but suffer null alleles, homoplasy, and complex and variable mutation processes that confound results (Defaveri et al., 2013; Putman & Carbone, 2014). The distribution of microsatellite markers genome‐wide is also unknown across many species. Still, they are likely not distributed evenly across the genome, potentially yielding a poorer representation of overall genetic variation than SNPs (Narum et al., 2013). Although microsatellites were once the most used marker in population genetics, SNPs are quickly replacing them in ecological, evolutionary, and conservation studies (Baruch & Weller, 2008). SNPs are biallelic and thus have a simpler mutation model but are less informative per locus, requiring more loci than would be needed for microsatellites to achieve the same statistical power (Helyar et al., 2011). SNPs also have lower error rates than microsatellites and, with next‐generation sequencing, have lower genotyping costs per marker (Morin et al., 2009; Weinman et al., 2015). However, SNPs are not immune to null alleles, especially when generated with restriction site‐associated DNA (RAD) sequencing approaches (Catchen et al., 2017; Lowry et al., 2017; Puritz et al., 2014). SNPs occur across the genome, providing a better representation of genome‐wide variation (Puckett & Eggert, 2016). Comparative assessments of the two markers found that SNPs outperformed microsatellites with estimates of genetic diversity and population structure (Morin et al., 2009; Muñoz et al., 2017) and performed equally or poorly with parentage analyses (Buchanan et al., 2017; Flanagan & Jones, 2019; Thrasher et al., 2018; Weinman et al., 2015). Nevertheless, microsatellites are still useful and can yield comparable results in population structure characterization and parentage inference (Liu et al., 2005; Väli et al., 2008). Although microsatellite versus SNP comparisons exist for genetic diversity estimates (Defaveri et al., 2013; Morin et al., 2004; Vali et al., 2008), population structure analyses (Helyar et al., 2011; Liu et al., 2005; Morin et al., 2009; Muñoz et al., 2017; Narum et al., 2008; Seddon et al., 2005), and parentage or pedigree inference (Baruch & Weller, 2008; Hauser et al., 2011; Kaiser et al., 2017; Labuschagne et al., 2015; Liu et al., 2017, Thrasher et al., 2018; Tokarska et al., 2009; Weinman et al., 2015), their relative performance in landscape genetics studies remains less understood (Hall & Beissinger, 2014; Puckett & Eggert, 2016). For instance, microsatellites outperform other codominant markers in assessing bottlenecks (Spencer et al., 2000), but it is not clear if this advantage persists over large numbers of SNP loci (Morin et al., 2012; Zimmerman et al., 2020). We need such direct comparisons to understand the biases, strengths, and weaknesses associated with different marker types for accurate data interpretation and to inform the adoption of new marker types in long‐term studies.

This study repeated microsatellite‐based population and landscape genetic analyses (Hauser et al., 2019; Hauser & Leberg, 2020) with SNPs developed from ddRAD sequencing (Peterson et al., 2012). This direct empirical comparison evaluated the relative performance of microsatellites and SNPs for landscape genetic analyses in bottlenecked populations, wherein higher statistical power will often be required to disentangle fine‐scale processes. The black‐capped vireo (*Vireo atricapilla*) source‐sink metapopulation in central Texas (Hauser et al., 2019; Hauser & Leberg, 2020; Walker et al., 2016) serves as an ideal bottlenecked system for marker comparison. The species is recovering from a demographic and a genetic bottleneck (Athrey et al., 2012; Grzybowski et al., 1994; McFarland et al., 2013) that resulted in small fragmented populations. Further, the species habitat range is highly fragmented through land conversion from their breeding habitat, scrub habitat, to agriculture and human development, resulting in most of the remnant population being restricted to protected habitats and military bases. The highest density of black‐capped vireos exists around Fort Hood in central Texas, where the species has been monitored carefully as a protected species (ESA Endangered from 1970–2018; Cimprich & Kostecke, 2006; Wilsey et al., 2014). The source‐sink system, in which Fort Hood broadly acts as a source to nearby small sink sites, comprises fragmented habitat patches driven by brown‐headed cowbird parasitism (Walker et al., 2016) and mediated by riparian corridors (Hauser & Leberg, 2020).

## 2 METHODS

**FIGURE 1:**
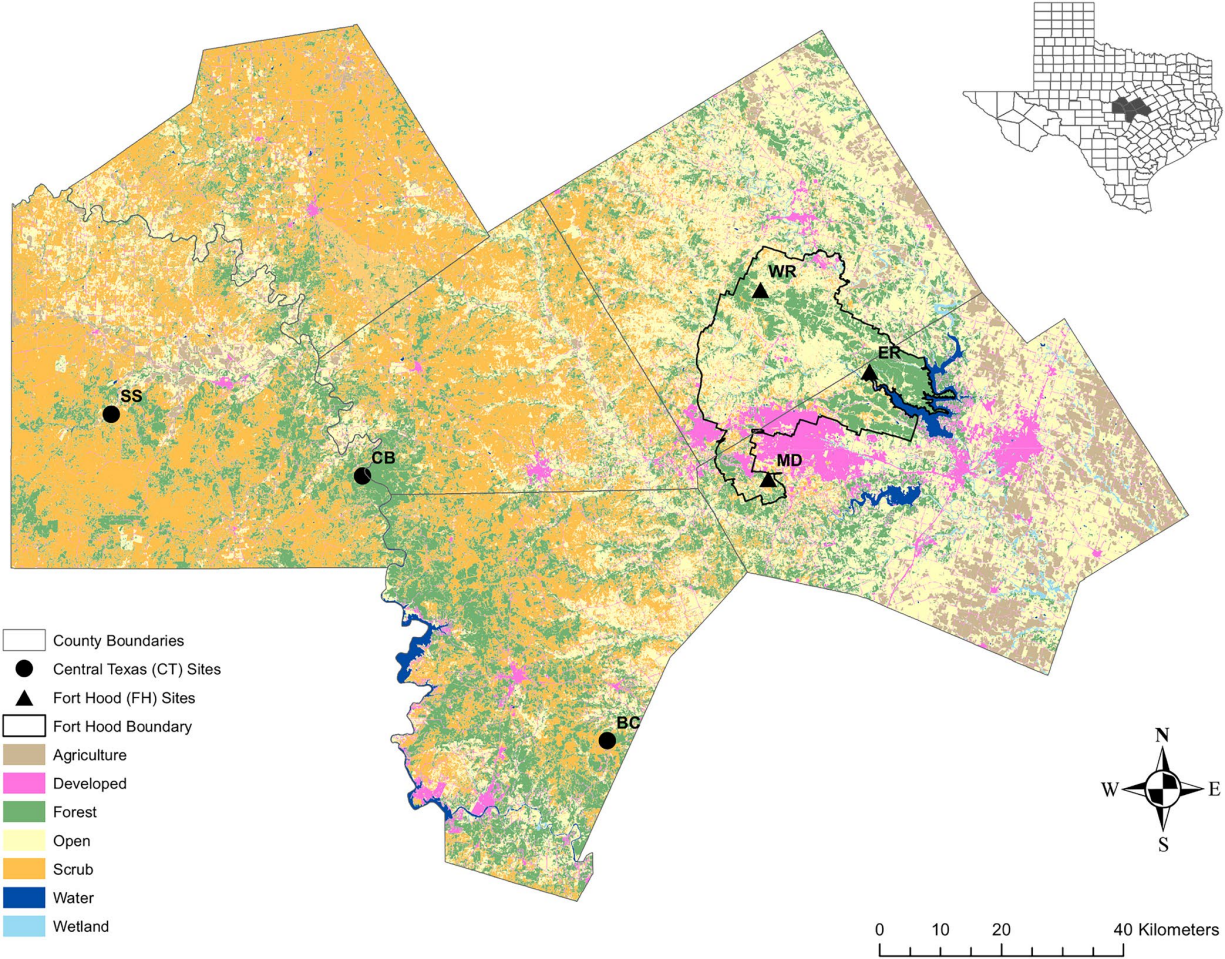
Black‐capped vireo study sites in central Texas (black circles) including Balcones Canyonlands (BC), Colorado Bend State Park (CB), San Saba Property (SS), and on Fort Hood (black triangles) including East Range combined (ER), Maxdale (MD), and West Range combined (WR). The six landscape cover types depicted as follows: agricultural croplands in brown, human development in magenta, forest in green, open habitat (including grazing lands) in yellow, scrub in orange, water bodies in navy blue, and wetlands in light blue

We collected DNA samples (toenail clips and/or pin feathers) from 338 black‐capped vireos from 6 sites in the breeding season (May–July) of 2014 and 2015 throughout central Texas, including Fort Hood [East Range (ER), Maxdale (MD), West Range (WR)], San Saba Property (SS), Balcones Canyonlands National Wildlife Refuge (BC), and Colorado Bend State Park (CB; see Hauser et al., 2019; Hauser & Leberg, 2020, for more details)]. We banded individuals with a unique U.S. Geological Survey band and a unique three‐color band combination, and sexed and aged using reliable molt limits (Hauser et al., 2019; Hauser & Leberg, 2020; Pyle, 1997). These sites span the source‐sink system identified by demography (Sources: ER, MD, and WR, Sinks: SS, BC and CB; Walker et al., 2016) and microsatellite analyses (Hauser et al., 2019; Hauser & Leberg, 2020; Figure 1). We extracted DNA from the samples using the Qiagen QIAamp Micro DNA Kit (Qiagen Inc, Hilden, Germany) following the protocol for isolation of genomic DNA from small volumes of blood. We also used these 338 samples in the microsatellite analysis using 12 species‐specific loci (Barr et al., 2007; Hauser et al., 2019; Hauser & Leberg, 2020), and to which we compared the results from the following SNP analysis.

We used 185 of the best quality black‐capped vireo samples for de novo SNP discovery and genotyping. We followed the ddRAD library preparation using the restriction enzymes speI and nlaIII for paired‐end 150‐bp reads (Peterson et al., 2012) and sequenced the libraries on an Illumina HiSeq 4000 lane. Library preparation, quality control, and sequencing were performed at the Texas A&M AgriLife Genomics core facility in College Station, Texas. Paired‐end sequence reads (total sequence reads = 802,466,640) were demultiplexed and filtered for poor quality using the process_radtags function in Stacks v2.0 (Rochette et al., 2019), retaining 1,960,156 total reads. We optimized parameters for the de novo pipeline, resulting in the following parameters for genotype calling: *m* = 3, *M* = 2, *n* = 1, *r* = 0.80, min_maf = 0.05. In optimization, we tested a range of parameters (*m* = 3–5; *M* = 2–6; *n* = 1–6; *r* = 0.8–0.9; min_maf = 0.01–0.05) and chose the combination that yielded the highest quantity of SNP loci per Paris et al. (2017). We filtered the dataset further in VCFtools for the minor allele count (mac = 3) and genotyping rate (80%, Danecek et al., 2011). For direct comparison, the microsatellite dataset (*n* = 338; Hauser et al., 2019; Hauser & Leberg, 2020) was subsampled to the same 185 individuals for which SNP data were produced. We performed all following analyses on both the subsampled microsatellite dataset and the SNP dataset generated herein. We designed the following analysis methods to parallel the microsatellite analyses described in Hauser et al. (2019) and Hauser and Leberg (2020) with minor modifications for large SNP datasets.

### 2.1 Population genetics

We tested loci per study site and samples for Hardy–Weinberg equilibrium (HWE) deviations and linkage disequilibrium (LD). Loci found to be in LD or deviating from HWE were omitted from further analysis. We calculated observed and expected heterozygosity (*H*_0_ and *H*_e_, respectively) using basic.stats function in hierfstat R package (v 3.5.0) and allelic richness (*A*_r_) using the allel.rich function in hierfstat R package (v 3.5.0) to estimate genetic diversity across the sites (Goudet, 2005). To evaluate how sites differed across these three metrics (*H*_0_, *H*_e_, and *A*_r_), we performed a randomized block ANOVA, blocking by locus, using the “aov” function in R with a post hoc Tukey HSD test using the TukeyHSD R function. In these and subsequent analyses, we corrected alpha levels for multiple comparisons using a standard Bonferroni correction (Hauser et al., 2019; Hauser & Leberg, 2020; Rice, 1989; Sethuraman et al., 2019). Wherever calculating p‐values with iterations was computationally impossible for our resources, we assessed significance using 95% confidence intervals (Altman & Krzywinski, 2016; Gardner & Altman, 1986).

We estimated population genetic differentiation (pairwise *F*_ST_) using the pairwise.WCfst function in R package hierfstat (v 3.5.0), estimating 95% confidence intervals with the boot.ppfst function in the same R package (Goudet, 2005). We assessed the population structure using the Bayesian clustering program STRUCTURE (v 2.3.4). We used the admixture model with population as a prior (i.e., LOCPRIOR function; Hubisz et al., 2009)) to determine the number of unique genetic clusters (k) present within our system, testing k values ranging from 1 to 6. We performed these runs with 10 iterations, 500,000 burn‐in period, and 500,000 MCMC (Monte Carlo Markov Chain) repetitions. We then submitted the STRUCTURE results to STRUCTURESELECTOR and used the Evanno and Puechmaille methods to determine k (Li & Liu, 2018; Puechmaille, 2016).

We used several approaches to investigate patterns of gene flow among the sites, specifically to determine if there was directional gene flow. Using GENECLASS (v 2.0), we detected first‐generation migrants using “L_home/L_max” likelihood ratio, the Paetkau et al. (1995) criterion, .01 allelic frequency, and .01 *p*‐value threshold. We used parentage assignments in CERVUS (v 3.0.7) to directly observe migration among sites (Kalinowski et al., 2007). For both the SNP and microsatellite datasets, we used the following simulation parameters for 10,000 simulated offspring based on censused black‐capped vireo demography (Cimprich & Kostecke, 2006; Walker et al., 2016, D. Cimprich, personal communication): number of candidate mothers = 414 (5.1% sampled) and candidate fathers = 581 (9.64% sampled), the proportion of loci typed = 0.90. We assigned 81 second‐year (SY) offspring to candidate mothers (*n* = 21) and fathers (*n* = 56), chosen by their relative age to a given SY offspring, using an SNP dataset with a 90% genotyping rate across the 178 individuals (N loci = 806), with a mean missingness of 8.3%. We additionally ran a small sensitive analysis to test ranges of parameters (number of offspring = 10,000–100,000; number of candidate mothers and father = 0.08–0.50; proportion of loci typed = 0.80–0.95). A more stringent genotyping rate was used for this analysis to avoid biases associated with missing data and parentage analyses (Hammerly et al., 2016) and to ensure that the program could accommodate the dataset (Kalinowski et al., 2007). Candidate parents needed to be sampled in the same year and in an age class old enough to feasibly produce SY offspring (after‐second‐year; ASY). For black‐capped vireos, SY individuals disperse and establish their first breeding territories, whereas older (ASY) individuals have strong site fidelity and remain in the same population for subsequent years. Therefore, we categorized offspring in populations different from their assigned parent as a migrant, whereas SY individuals found to be in the same population as their parents were considered residents.

### 2.2 Landscape genetics

For all landscape genetics analyses, we used the population‐level proportion of shared alleles (Dps) as a metric of gene flow (pairwise. PropShared function in R package PopGenReport; Adamack & Gruber, 2014; Gruber & Adamack, 2017) as Dps is more directly related to gene flow than other metrics of genetic differentiation (Landguth et al., 2010). We tested for isolation by distance at individual and population levels using the mantel.randtest function in the R package adegenet (Jombart, 2008).

We used the same between‐site and at‐site predictor variable database as Hauser and Leberg (2020), including elevation, Euclidean distance, water, development, forest, scrub, open, agriculture, riparian, the proportion of scrub habitat, and brown‐headed cowbird (BHCO) management at the sites. Between‐site variables (elevation, Euclidean distance, water, development, forest, scrub, open, agriculture, and riparian) were transformed into resistance surfaces in CIRCUITSCAPE (McRae, 2006). We optimized the valuation of each resistance surface (see Hauser and Leberg (2020) for more details on optimization) using a linear mixed‐effects model (R package lme4; Bates et al., 2015) with Dps as the response variable, each resistance value as the fixed effect, and site as the random effect. Only the optimized resistance values for a given variable, the value with the lowest AICc score via the univariate linear mixed‐effects models, were used in subsequent hypotheses testing.

To investigate how landscape features influence gene flow in this system, we used a multivariate linear mixed‐effects model approach using candidate models driven by *a priori* hypotheses (Table 1). All candidate models were checked for multicollinearity using a variance inflation factor (VIF) threshold of 4 before fitting the models. We used the linear mixed‐effects models in the R package lme4 using the full maximum likelihood with D_ps_ as the response variable, landscape features as fixed effects, and site as the random effect (Bates et al., 2015). We evaluated our candidate models with AIC_c_, ΔAIC_c_, and AIC_c_ weights (R package GeNetIt). We considered models with a ΔAIC_c_ < 2 to be competitive (Burnham & Anderson, 2002). Across all methods, we compared results from the SNP data, the subsampled microsatellite data, and the complete microsatellite data (*n* = 338) presented in Hauser et al. (2019) and Hauser and Leberg (2020).

**TABLE 1:** Multivariate candidate models with predicted relationship with gene flow in parentheses (e.g., (−)Agriculture denotes a negative relationship between agriculture and gene flow), and the *a priori* hypothesis (rationale) for landscape genetic analyses for the black‐capped vireo source‐sink system

Candidate model	Rationale
(−)Ag + (−)Dev + (−)Open	Human‐caused habitat fragmentation
(+)Elevation + (+)Scrub	Breeding habitat and associated high elevation
(+)Elevation + (+)Riparian	Riparian areas (potential corridors) and associated breeding habitat elevation
(+)Riparian + (−)Ag + (−)Open	Riparian areas and habitat fragmentation
(+)Riparian + (−)Water + (−)Ag + (−)Open	Riparian areas, waterways, and habitat fragmentation
(+)Riparian + (−)Water + (+)Scrub	Riparian areas, waterways, and breeding habitat
(+)From_Scrub + (+)From_CowbirdControl	Site productivity (large habitat patch and low nest parasitism)
(−)Ag + (−)Dev + (−)Open + (+)From_CowbirdControl	Habitat fragmentation and site productivity due to nest parasitism
(+)Riparian + (−)Water + (−)Ag + (−)Open + (+)From_Scrub + (+)From_CowbirdControl	Habitat fragmentation, riparian areas, and site productivity
(+)Riparian + (−)Water + (+)Scrub + (+)From_Scrub + (+)From_CowbirdControl	Riparian areas, waterways, breeding habitat, and site productivity

Note

These set of a priori candidate models were used for both microsatellite and SNP datasets.

Abbreviations: Ag, agriculture; Dev, developed; Distance, isolation by distance; From_CowbirdControl the level of Brown‐headed Cowbird control at the emigration site, From_Scrub the area of scrub habitat at the emigration site.

## 3 RESULTS

### 3.1 Population genetics

After filtering, the genomic dataset included 11,507 SNP loci for 178 individuals (Table 2), with a mean coverage of 18.2× and a mean missingness of 15.2%. The microsatellite dataset was also subsampled to the same 178 individuals. We found no deviations from HWE or LD for both datasets after a Bonferroni correction at any of our study sites.

**TABLE 2:** Summary of sample size, expected heterozygosity (*H*_e_), observed heterozygosity (*H*_0_), and allelic richness (*A*_r_) over 12 microsatellite loci

Pop	*n*	*H*_e_	*H*_0_	*A*_r_
Microsatellites				
SS	11	0.836 (0.794–0.877)	0.689 (0.626–0.752)	7.23 (6.64–7.82)
BC	12	0.805 (0.763–0.846)	0.785 (0.722–0.848)	6.30 (5.70–6.89)
CB	10	0.837 (0.795–0.878)	0.764 (0.701–0.827)	7.19 (6.60–7.78)
ER	39	0.819 (0.778–0.861)	0.697 (0.634–0.760)	7.91 (7.32–8.50)
MD	11	0.808 (0.766–0.849)	0.683 (0.620–0.746)	7.45 (6.86–8.04)
WR	95	0.789 (0.748–0.831)	0.508 (0.445–0.571)	6.70 (6.11–7.29)
SNPs				
SS	11	0.171 (0.170–0.173)	0.173 (0.171–0.175)	1.310 (1.306–1.313)
BC	12	0.173 (0.171–0.175)	0.159 (0.157–0.161)	1.316 (1.313–1.32)
CB	10	0.171 (0.169–0.173)	0.153 (0.151–0.154)	1.308 (1.305–1.312)
ER	39	0.176 (0.174–0.178)	0.170 (0.168–0.172)	1.330 (1.326–1.333)
MD	11	0.174 (0.172–0.175)	0.160 (0.158–0.162)	1.316 (1.313–1.319)
WR	95	0.174 (0.172–0.176)	0.162 (0.160–0.164)	1.328 (1.325–1.331)

Note

95% confidence intervals are in parentheses. The subsampled microsatellite analysis is featured in the top panel (*n* = 178) and the SNP analysis is featured in the bottom panel (*n* = 178).

Abbreviations: BC, Balcones Canyonlands National Wildlife Refuge; CB, Colorado Bend State Park; ER, East Range (Fort Hood); MD, Maxdale (Fort Hood); SS, San Saba Property; WR, West Range (Fort Hood).

**FIGURE 2:**
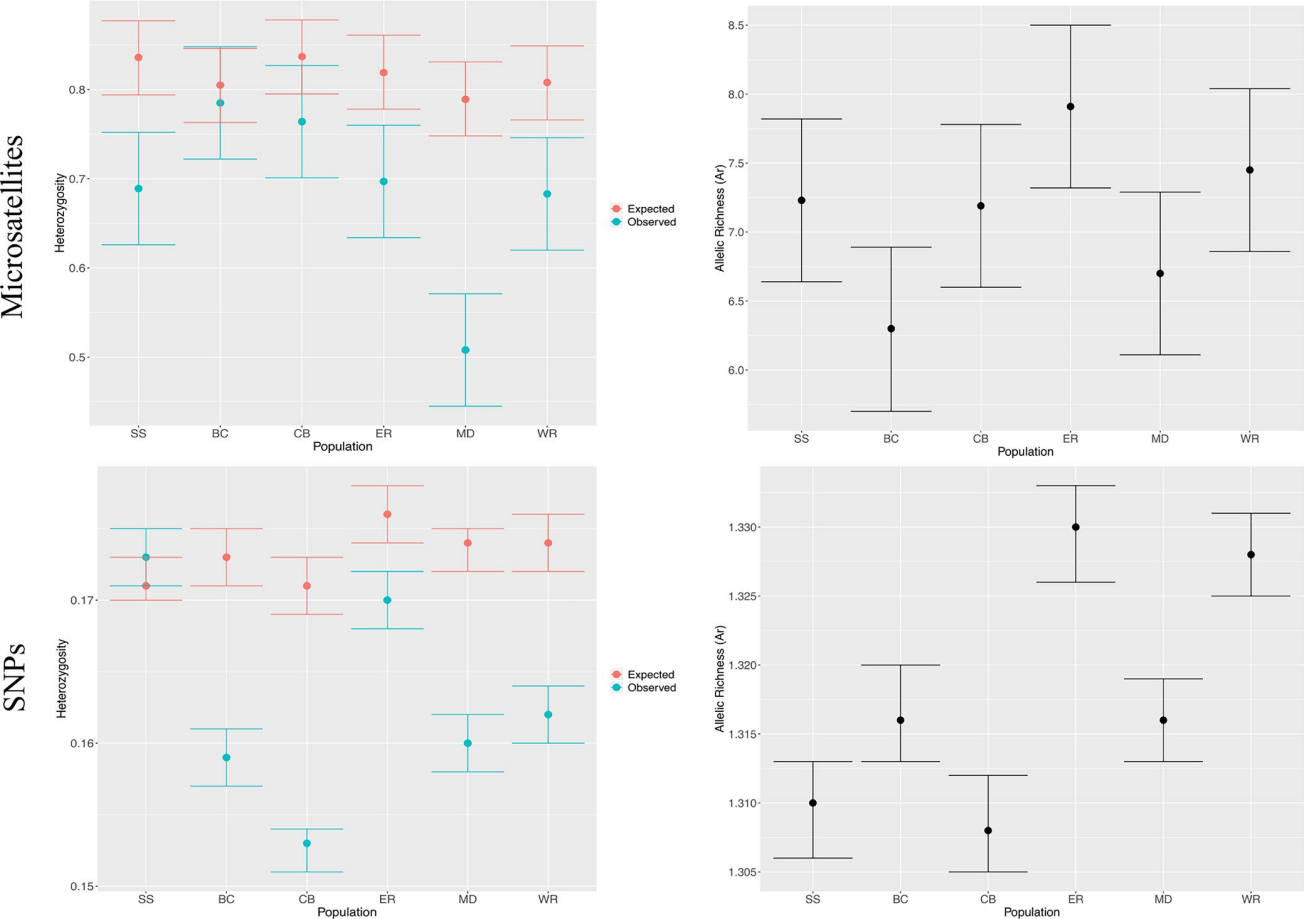
Genetic diversity estimates (dots) with 95% confidence intervals (error bars) per black‐capped vireo population: BC, CB, ER, MD, SS, WR. Observed and expected heterozygosity (blue and orange, respectively) per population in the left panel and allelic richness (*A*_r_, in black) per population on the right panel. Estimates in which their 95% confidence intervals overlap are not statistically different

For the microsatellites, we found significant differences for *H*_0_ and *A*_r_ among sites (*p* < .001) but not for *H*_e_ (*p* = .549). All sites, except BC and CB, had significantly lower *H*_0_ than *H*_e_ (Figure 2, top panel). MD was the only population with an estimate of *H*_0_ that was significantly different from the other 6 sites. Across sites, there were no significant differences in *H*_e_ or *A*_r_, with the exception of a significant difference in *A*_r_ between BC and ER. The full microsatellite data from Hauser et al. (2019) and Hauser and Leberg (2020) exhibited no differences in any of the genetic diversity metrics across sites. For the SNPs, we found significant differences for *H*_0_, *H*_e_, and *A*_r_ among sites (*p* < 0.001; Table 2, Figure 2, bottom panel). All sites, except SS, had significantly lower *H*_0_ than *H*_e_. There were significant differences in *H*_0_ across sites in three broad groupings: CB had the lowest *H*_0_ values; BC, MD, and WR had the intermediate values; and ER and SS had the highest *H*_0_ values. There were also significant differences in *A*_r_ among sites, namely, ER and WR were significantly higher than the rest of the sites (Figure 2). Values for all genetic diversity metrics and their variances calculated using microsatellites were much higher than those using SNPs (Table 2).

**FIGURE 3:**
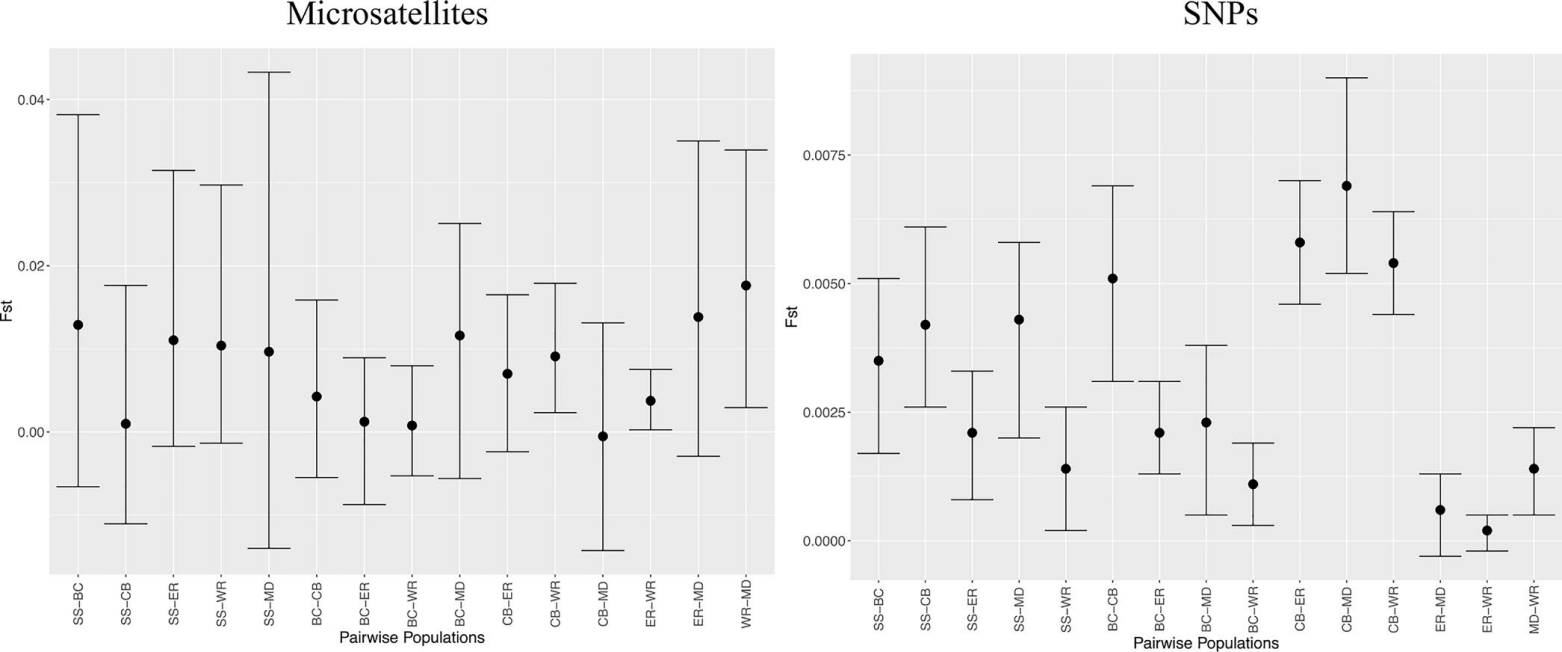
Pairwise *F*_ST_ estimates (dots) with 95% confidence intervals (error bars) between black‐capped vireo populations: BC, CB, ER, SS, MD, WR. Estimates that overlap with 0 are not statistically significant and estimates in which 95% confidence intervals overlap are not statistically different from one another

**FIGURE 4:**
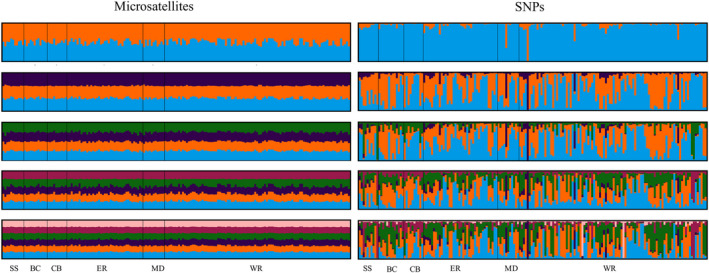
Weak to no population structuring among Blackbcapped vireo populations (BC, CB, SS, ER, MD, and WR). STRUCTURE barplots for *k* values (number of unique clusters) 2 through 4. Each vertical line represents the genetic signature of an individual with colors representing each cluster

As expected, the full microsatellite dataset showed that most genetic differentiation was between central Texas sites and Fort Hood sites (Table 3; Hauser et al., 2019; Hauser & Leberg, 2020). WR and ER, Fort Hood sites, were the only significantly differentiated sites relative to the rest of the source‐sink system. Likewise, the complete microsatellite dataset showed that most differentiation was between central Texas sites and Fort Hood sites (Hauser et al., 2019; Hauser & Leberg, 2020). All pairwise *F*_ST_ values based on SNPs were statistically significant, except between ER and WR (Figure 3). We found the greatest population differences between central Texas site CB and the Fort Hood sites. Central Texas sites were differentiated from WR, ER, and MD (increasing in that order). There was no overall pattern that central Texas sites were more similar to other central Texas sites than with Fort Hood sites or vice versa. *F*_ST_ values calculated with microsatellites were an order of magnitude higher than those calculated with SNPs. The Puechmaille method, which accounts for uneven sampling (Li & Liu, 2018; Puechmaille, 2016), showed two unique genetic clusters for the microsatellite data and the full microsatellite dataset (Hauser et al., 2019; Hauser & Leberg, 2020), whereas the same approach using SNP markers identified only one cluster. All sets of STRUCTURE barplots based on SNPs showed no population subdivision and considerable mixing, regardless of marker across k values ranging from 2 to 6 (Figure 4; Hauser et al., 2019; Hauser & Leberg, 2020).

**TABLE 3:** Genetic differentiation between sites sampled for black‐capped vireos

	SS	BC	CB	ER	MD	WR
Microsatellites						
SS	–	−0.007 to 0.038	−0.011 to 0.018	−0.002 to 0.031	−0.001 to 0.030	−0.014 to 0.043
BC	0.013	–	−0.005 to 0.016	−0.009 to 0.009	−0.005 to 0.008	−0.006 to 0.025
CB	0.001	0.004	–	−0.002 to 0.017	**0.002 to 0.018**	−0.014 to 0.013
ER	0.011	0.001	0.007	–	0.000 to 0.008	−0.003 to 0.035
MD	0.010	0.001	**0.009**	0.004	–	**0.003 to 0.034**
WR	0.010	0.012	−0.001	0.014	**0.018**	–
SNPs						
SS	–	**0.0017 to 0.0051**	**0.0026 to 0.0061**	**0.0008 to 0.0033**	**0.0020 to 0.0058**	**0.0002 to 0.0026**
BC	**0.0035**	–	**0.0031 to 0.0069**	**0.0013 to 0.0031**	**0.0005 to 0.0038**	**0.0003 to 0.0019**
CB	**0.0042**	**0.0051**	–	**0.0046 to 0.0070**	**0.0052 to 0.0090**	**0.0044 to 0.0064**
ER	**0.0021**	**0.0021**	**0.0058**	–	−0.0003 to 0.0013	−0.0002 to 0.0005
MD	**0.0043**	**0.0023**	**0.0069**	0.0006	–	**0.0005 to 0.0022**
WR	**0.0014**	**0.0011**	**0.0054**	0.0002	**0.0014**	–

Note

Pairwise *F*_ST_ values are depicted on the lower left and 95% confidence intervals are shown on the upper right. Values that are significant, i.e., the 95% confidence interval overlaps with 0, are in bold. The subsampled microsatellite analysis is featured in the top panel (*n* = 178) and the SNP analysis is featured in the bottom panel (*n* = 178).

Abbreviations: BC, Balcones Canyonlands National Wildlife Refuge; CB, Colorado Bend State Park; ER, East Range (Fort Hood); MD, Maxdale (Fort Hood); SS, San Saba Property; WR, West Range (Fort Hood).

Using the subsampled microsatellite data, we found 125 migrants, 25 detected in central Texas and 100 detected in Fort Hood. Of these detected migrations, 79 were between central Texas and Fort Hood, 9 were among central Texas sites, and 37 were among Fort Hood sites. Similar to the SNP data, migrants found in central Texas sites comprised a substantially greater portion of the estimated census population size (13.2–20.5%) than those in Fort Hood sites (1.8–3.0%). Hauser et al. (2019) and Hauser and Leberg (2020) detected fewer migrants overall (*n* = 22), but similar patterns in proportion of migrants in sites were found. Using SNPs, we found 82 migrants, with 33 detected in central Texas sites and 49 detected in Fort Hood sites using GENECLASS2 (Table 4). All migrants detected were from WR. Migrants in central Texas sites comprised a much larger proportion of the total population (14.7–29.5%) than those detected in Fort Hood (<1%–6.9%). Regardless of dataset, proportions of migrants in central Texas sites were an order of magnitude higher than those in Fort Hood.

**TABLE 4:** Total number of detected 1st generation migrants per site (# M), proportion of detected migrants per site of all total migrants (% M), estimated census population size (N; Cimprich et al., 2009; Walker et al., 2016), and proportion of detected migrants of total censused population size per site (% N) (GENECLASS2)

Population	# M	% M	N	% N
Microsatellites				
Central Texas				
SS	8	6.4	39	20.5
BC	8	6.4	44	18.2
CB	9	7.2	68	13.2
Fort Hood				
ER	30	24.0	993	3.0
MD	10	8.0	160	6.3
WR	60	48.0	3292	1.8
SNPs				
Central Texas				
SS	10	12.2	39	25.6
BC	13	15.9	44	29.5
CB	10	12.2	68	14.7
Fort Hood				
ER	38	46.3	993	3.8
MD	11	13.4	160	6.9
WR	0	0.0	3292	0.0

Note

The subsampled microsatellite analysis is featured in the top panel (*n* = 178), and the SNP analysis is featured in the bottom panel (*n* = 178).

Abbreviations: BC, Balcones Canyonlands National Wildlife Refuge; CB, Colorado Bend State Park; ERc, East Range (Fort Hood); MD, Maxdale (Fort Hood); SS, San Saba Property; WRc, West Range (Fort Hood).

In CERVUS, the microsatellite analysis assigned 20 parent–offspring pairs at the 95% confidence interval (Table 5). We identified most offspring assigned to parents as migrants (*n* = 16), of which most were from Fort Hood (*n* = 14). We found directional migration from Fort Hood to central Texas (*n* = 4) compared with central Texas to Fort Hood (*n* = 1). The full microsatellite dataset assigned more parent–offspring pairs (*n* = 21) at the 95% confidence interval (Hauser et al., 2019; Hauser & Leberg, 2020) and indicated similar patterns of directional migration from Fort Hood to central Texas as the subsampled dataset. The SNP‐based analysis did not assign any candidate parents to offspring across any of the tested parameter settings.

**TABLE 5:** Number of offspring assigned to candidate offspring (CERVUS) and designated as migrants or residents (N) using the subsampled microsatellite data

Movement	Movement	%	% Total
Migrants	16	–	80
FH to CT	4	25	20
CT to FH	1	6	5
CT to CT	1	6	5
FH to FH	10	63	50
Residents	4	–	20
CT	0	0	0
FH	4	100	20

Note

Directional movement between regions (Fort Hood (FH) and central Texas (CT) (e.g., FH to CT denotes movement from Fort Hood to central Texas), percentage of each subcategory, migrants or residents (%), and percentage of total number of assigned offspring (% total) are also shown. Assignment data using SNP data not shown as there were no successful parent–offspring assignments.

### 3.2 Landscape genetics

**FIGURE 5:**
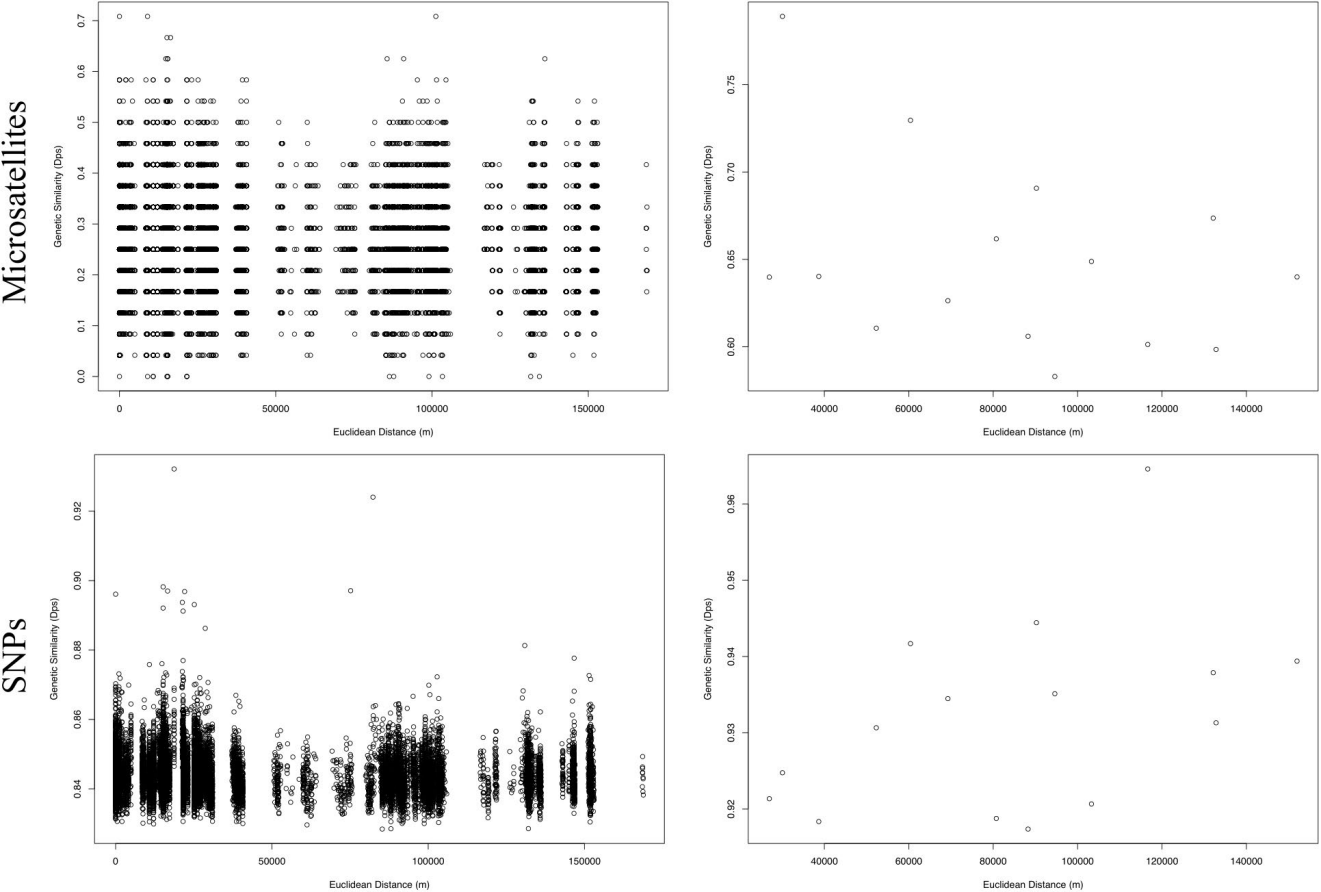
No signature of isolation by distance at an individual (left panel) or population level (right panel). The relationship between genetic similarity (proportion of shared alleles; Dps) on the y‐axis and Euclidean distance (in meters; m) on the *x*‐axis

For all datasets, there was no evidence of isolation by distance at either a population or individual level (microsatellites: *p* = .92, *p* = .492, respectively; SNPs: *p* = .092, *p* = .946, respectively; Hauser et al., 2019; Hauser & Leberg, 2020; Figure 5). The null model of isolation by distance in our multivariate linear mixed‐effects models only had low support via AIC evaluation for the SNPs and the full microsatellite dataset.

For the subsampled microsatellite data, 11 of the 20 candidate models had ΔAIC_c_ < 2, including the null model, indicating a substantial loss of power using microsatellites with this reduced sample size (Table 6). Top models with ΔAIC_c_ < 2 for the SNP dataset were “riparian + water + scrub” and “development” (Table 6). The top models from Hauser and Leberg (2020) were “agriculture + development + open” and “riparian + agriculture + open.” Hauser and Leberg (2020) indicated that agriculture and riparian areas facilitated gene flow, whereas development and open habitat impeded gene flow. From our analyses, riparian areas facilitated gene flow (ß = 0.022, respectively) and scrub habitat facilitated gene flow (ß = 0.0087), whereas water impeded gene flow (ß = −0.033) and development impeded gene flow (ß = −0.0038). Common variables across the two datasets were riparian and development; the relationships of habitat with gene flow were similar.

**TABLE 6:** Summary of linear mixed‐effects models results including AICc, delta AICc values and AICc weights for the candidate models

Microsatellites				SNPs			
Model	AICc	ΔAICc	AICw	Model	AICc	ΔAICc	AICw
**Open2**	**−118.44**	**0.00**	**0.10**	**Wetlands001 + Water100 + Scrub05**	**−265.32**	**0.00**	**0.40**
**Wetland001**	**−118.32**	**0.12**	**0.10**	**Dev100**	**−264.76**	**0.56**	**0.31**
**Ag100**	**−118.28**	**0.15**	**0.09**	Wetlands001 + Water100 + Ag100 + Open2	−263.19	2.13	0.14
***Euclidean distance (Null)***	***−118.24***	***0.20***	***0.09***	Ag100 + Dev100 + Open2	−261.16	4.16	0.05
**Elevation**	**−118.24**	**0.20**	**0.09**	Water100 + Wetland001 + Scrub05 + From_BHCO + From_Scrub	−261.14	4.18	0.05
**Water100**	**−118.19**	**0.25**	**0.09**	Forest001	−259.87	5.44	0.03
**Scrub05**	**−118.06**	**0.38**	**0.08**	Water100 + Wetland001 + Ag100 + Open2 + From_Scrub + From_BHCO	−258.98	6.34	0.02
**Dev100**	**−117.76**	**0.68**	**0.07**	Water100	−254.64	10.68	0.00
**Forest001**	**−117.39**	**1.05**	**0.06**	Wetland001 + Ag100 + Open2	−254.42	10.90	0.00
**Elevation + Wetland001**	**−116.63**	**1.81**	**0.04**	Open2	−253.91	11.41	0.00
**Elevation + Scrub05**	**−116.61**	**1.82**	**0.04**	Ag100	−253.79	11.52	0.00
Wetlands001 + Water100 + Scrub05	−116.38	2.06	0.04	Wetland001	−252.76	12.56	0.00
Ag100 + Dev100 + Open2	−115.69	2.75	0.03	*Euclidean Distance (Null)*	*−252.73*	*12.59*	*0.00*
From_BHCO	−114.92	3.52	0.02	Scrub05	−252.42	12.89	0.00
From_Scrub	−114.86	3.58	0.02	Elevation + Wetland001	−250.93	14.39	0.00
Wetland001 + Ag100 + Open2	−114.31	4.13	0.01	Elevation + Scrub05	−250.83	14.49	0.00
Wetlands001 + Water100 + Ag100 + Open2	−114.30	4.14	0.01	Elevation	−249.99	15.33	0.00
From_Scrub + From_BHCO	−112.86	5.58	0.01	From_Scrub	−231.15	34.17	0.00
Water100 + Wetland001 + Scrub05 + From_BHCO + From_Scrub	−112.24	6.20	0.00	From_BHCO	−231.14	34.18	0.00
Water100 + Wetland001 + Ag100 + Open2 + From_Scrub + From_BHCO	−110.14	8.30	0.00	From_Scrub + From_BHCO	−229.09	36.23	0.00

Note

Bolded values indicate delta AICc values <2. Scores for our null model, isolation by distance are italicized. Numbers next to variable names (e.g., Ag100) indicate the optimized value parameterized for the associated variable in CIRCUITSCAPE. The subsampled microsatellite analysis is featured in the left panel (*n* = 178), and the SNP analysis is featured in the right panel (*n* = 178).

Abbreviations: Ag, agriculture; Dev, developed; Distance, isolation by distance; From_BHCO, the level of BHCO control at the emigration site; From_Scrub, the amount of scrub habitat at the emigration site.

## 4 DISCUSSION

There was overarching agreement in the inferences based on SNP and microsatellites datasets; both types of markers detected the black‐capped vireo source‐sink system with WR and ER as putative source sites and the remaining sites as sinks. Although we found agreement between the two marker types in the overall patterns (i.e., asymmetrical gene flow, weak structuring, and admixture), specific results differed between the datasets. Among population genetic estimates, we found statistically significant heterozygosity deficiencies in many sites, higher allelic richness in ER and WR, statistically significant pairwise *F*_ST_ values among population pairs, and detection of first‐generation migrants. The microsatellite analyses found fewer differences in heterozygosity or allelic richness among sites, and few pairwise *F*_ST_ tests were significant. The SNP dataset was unsuccessful in reconstructing parentage, potentially due to insufficient power associated with biallelic markers compared with multiallelic microsatellites. For the landscape genetic results, the subsampled microsatellite data failed to identify any relevant top models. Although the SNP and the complete microsatellite datasets (Hauser & Leberg, 2020) yielded two common landscape variables (riparian and developed), the top models from these datasets were not in agreement for other variables.

Many of the discrepancies between the SNP and microsatellite results can be attributed to the higher loci number and thus greater statistical power associated with SNP datasets. A large number of biallelic SNPs deflate and restrict the range of heterozygosity, allelic richness, and *F*_ST_ values compared with those of multiallelic microsatellites (Weir & Hill, 2002). Regardless of the marker used, most black‐capped vireo sites had lower heterozygosity than expected, and putative source sites ER and WR had significantly higher allelic richness than the rest of the sites, but SNPs yielded fewer overlapping and smaller confidence intervals with these genetic diversity estimates (higher precision). Our ability to detect fine‐scale genetic differentiation using SNPs improved with greater pairwise differentiation (*F*_ST_). Previous studies have found that SNPs are more accurate at estimating genetic diversity metrics (Muñoz et al., 2017; Seddon et al., 2005) and genetic structuring (Liu et al., 2005; Morin et al., 2009; Seddon et al., 2005). However, the Bayesian clustering approach STRUCTURE was unable to detect fine‐scale population structuring for either marker. This software has been found to perform poorly with fine‐scale structure (Janes et al., 2017) and likely could not disentangle small levels of structuring in this metapopulation. Black‐capped vireos show strong fine‐scale structuring (Athrey et al., 2015), which may contribute to the results here. With high levels of gene flow characteristic of a metapopulation (Edelaar & Bolnick, 2012), we would not expect to see strong genetic structuring in the black‐capped vireo source‐sink system.

The inability to reconstruct parentage using SNPs in the present study may be due to the lower information content of SNP markers than the multiallelic microsatellites. Several studies have shown that microsatellites outperform SNPs with parentage analyses because of their high polymorphism information content per locus (Defaveri et al., 2013; Weinman et al., 2015). Further, parentage depends primarily upon heterozygosity values to reconstruct relationships. As SNPs have lower heterozygosity values, they consequently lose the ability to reconstruct relationships (Kaiser et al., 2017; Morin et al., 2004; Tokarska et al., 2009; Weinman et al., 2015). Morin et al. (2004) indicated that a heterozygosity minimum of 0.20 is required for paternity exclusion analyses, but Blouin et al. (1996) have found that even higher values (*H*_e_ = 0.60 – 0.75) would be necessary to reconstruct 1st‐order relationships accurately. As the maximal heterozygosity value possible with SNP loci is 0.50 (Tokarska et al., 2009), it is unsurprising that SNPs often provide insufficient information to reconstruct parentage. Currently, there is no consensus on the superior marker for parentage analyses (Flanagan & Jones, 2019; Thrasher et al., 2018) as several interacting factors affect the ability to reconstruct parentage, such as missing data and the number of markers available. Parentage is highly sensitive to missing data, whereas population genetic analyses (genetic diversity and population structure) can be more robust to relatively low levels of missing genotype data (Hammerly et al., 2016; Kaiser et al., 2017; Shafer et al., 2017). The high number of loci associated with SNP genomic markers is often the reasoning upholding SNPs’ outperformance relative to microsatellites (Hess et al., 2011; Zimmerman et al., 2020). Our other population genetic results support the assertion that although SNP data have substantially higher statistical power than microsatellite data, these benefits do not necessarily extend to parentage analysis due to the low heterozygosity values (0.153–0.176). For this system and many other nonmodel systems in which low genetic diversity and/or bottlenecks have occurred (i.e., threatened or endangered species), markers with high information content such as microsatellites, microhaplotypes, or haplotypes may be more useful for parentage analyses than SNPs (Baetscher et al., 2018; Jones et al., 2009).

Despite SNPs’ purported higher resolution into population genetic processes, as seen here and many other comparisons (Kaiser et al., 2017; Kleinman‐Ruiz et al., 2017; Seddon et al., 2005), significant findings do not necessarily translate to biologically relevant differences. Statistically significant differences found in genetic diversity (heterozygosity and allelic richness) and structure metrics (*F*_ST_) among the black‐capped vireo study sites were extremely small (on the order of thousandths) and may lack biological significance. When calculating population genetic metrics, large SNP datasets, such as ours, increase the chance of statistically significant results (using p‐values or 95% confidence intervals) and Type I error of results (Wigginton et al., 2005).

This study serves as one of the first direct marker comparisons (others include Hall and Beissinger, (2014) and Puckett and Eggert, (2016)) in a landscape genetic context showing varying results between SNPs and microsatellites. Neither SNPs nor microsatellites found any evidence for isolation by distance, as would be expected for a metapopulation with considerable admixture as seen here (Gaggiotti, 1996; Jenkins et al., 2010). Isolation by distance as a model in the linear mixed model analysis consistently showed low support for the SNP and full microsatellite datasets. Although both datasets identified overlapping landscape variables (riparian and human development), we found some discrepancies between the top models of each marker. The landscape genetic analyses with SNPs identified additional landscape variables to the full microsatellite dataset (Hauser & Leberg, 2020): scrub and water, although not identifying agriculture and open habitat in top models. Scrub is the breeding habitat of the black‐capped Vireo vnd would be expected as both a top model and a vital landcover type for facilitating dispersal. Large water bodies in this area, not associated with riparian areas, are likely driving the negative relationship between water and gene flow. The full microsatellite analysis found that agricultural areas facilitated gene flow, opposite to predictions and black‐capped vireo observations. The subsampled microsatellite data did not yield any significant top models as it indicated that 11 of the 20 *a priori* models were equally informative and as equally as informative as the null model, isolation by distance, for which formal testing showed no relationship. Although we cannot say which marker produces the more accurate results in this system, landscape genetic analyses using microsatellites require higher sampling than SNP analyses. Nevertheless, it is promising that both markers identify similar landscape variables (riparian and scrub) that have been corroborated by observational and telemetry data (Dittmar et al., 2014).

Formal comparisons between SNPs and microsatellites have been lacking in landscape genetics, especially in populations recovering from bottlenecks. Although our comparison helps fill said gap, it is not satisfactory in a complete investigation of marker performance. Genetic distance metrics are often more precisely estimated using SNPs (Morin et al., 2009; Muñoz et al., 2017) and therefore could yield more accurate landscape genetic inferences. However, metrics such Dps as used in the present study have not been used in formal comparison and simulation studies. We need further investigations in landscape genetics to understand the respective accuracy and precision of microsatellites and SNPs, especially as many contemporary landscape genetics research is being done with one marker or the other.

We show that overall SNP and microsatellite data can infer similar biological processes and patterns. Microsatellites can still be used for a wide variety of population or conservation questions, despite an extensive adoption of genomics techniques in the field. We especially want to add our voice to the assertion for systems with existing or legacy microsatellite panels, in which development of new markers would be costly, piecewise genotyping is commonplace (as found in management), or where bioinformatics expertise or computational power is not accessible (Flanagan & Jones, 2019). In species with low genetic diversity or that have experienced bottlenecks, especially prevalent in conservation genetics, multiallelic markers, such as microsatellites, could provide the necessary power in parentage analyses when SNPs cannot. Nevertheless, in developing new molecular markers for a population genetic study, SNPs are less expensive per locus than microsatellites and have substantially more statistical power than microsatellites for most comparisons, yielding a cost‐effective approach over microsatellites (Flanagan & Jones, 2019). SNPs also allow for investigation into adaptive variation with loci under selection whereas microsatellites cannot (Ahrens et al., 2018; Flanagan & Jones, 2019; Helyar et al., 2011). A third‐choice researchers should consider is the microhaplotype, a multiallelic marker produced through next‐generation techniques, which yields a higher number of loci than microsatellites and is randomly distributed throughout the genome (Baetscher et al., 2018). We urge researchers to thoroughly consider the utility of each marker based on their system and urge reviewers and editors not to disregard research using microsatellites. This comparison serves as an illustration of such a case where microsatellite and SNP results converge in conclusions and microsatellites still maintain a function in population genetics.

## CONFLICT OF INTEREST

The authors have no conflicts of interest to report.

## AUTHOR CONTRIBUTION

**Samantha S. Hauser:** Conceptualization (equal); Data curation (lead); Formal analysis (lead); Methodology (equal); Software (lead); Validation (lead); Writing‐original draft (lead). **Giridhar Athrey:** Conceptualization (equal); Formal analysis (supporting); Methodology (supporting); Supervision (supporting); Validation (supporting); Writing‐review & editing (supporting). **Paul L. Leberg:** Conceptualization (equal); Funding acquisition (lead); Project administration (lead); Resources (lead); Supervision (lead); Writing‐review & editing (supporting).

## Data Availability

Microsatellite primer Genbank accession numbers: EF363782‐EF36378295. Genotype data in VCF and genepop file formats, environmental data, and code are be available on Dryad (https://doi.org/10.5061/dryad.n5tb2rbwk).
